# Supplementary material for: Posttraumatic Stress Symptoms and Attitudes toward the China Eastern Airlines Plane Crash in Transportation Students
Source: Int J Environ Res Public Health. 2022 Sep 10;19(18):11400. doi: 10.3390/ijerph191811400 (PMC9517083; doi:10.3390/ijerph191811400)
Supplement: Supplementary file 1 [file ijerph-19-11400-s001.zip › ijerph-1898369-supplementary.pdf]

Supplementary Appendix

Table of Contents

Table S1. Prevalence of depressive, anxiety, and insomnia symptoms, and PTSS among participants by groups and gender

Table S2. Prevalence of depressive, anxiety, and insomnia symptoms among participants by severity

Figure S1. Scatter plots of the linear correlations between PHQ-9, GAD-7, ISI, fatigue, and PCL-C scores of the general public sample

Figure 1S_a. Correlation between PHQ-9 and PCL-C scores

Figure 1S_b. Correlation between GAD-7 and PCL-C scores

Figure 1S_c. Correlation between ISI and PCL-C scores

Figure 1S_d. Correlation between fatigue and PCL-C scores

Table S1. Prevalence of depressive, anxiety, and insomnia symptoms, and PTSS among participants by groups and gender

Variables	Total	Male	Feamle	χ^2	P
	Number (%, 95CI)				
**Attendant students**	***n*=183**	***n*=35**	***n*=148**		
Depressive symptoms	95 (51.9, 44.6-59.2)	18 (51.4, 34.0-68.8)	77 (52.0, 43.9-60.2)	0.004	0.949
Anxiety symptoms	74 (40.4, 33.3-47.6)	14 (40.0, 22.9-57.1)	60 (40.5, 32.5-48.5)	0.003	0.953
Insomnia symptoms	46 (25.1, 18.8-31.5)	13 (37.1, 20.3-54.0)	33 (22.3, 15.5-29.1)	3.315	0.069
PTSS	23 (12.6, 7.7-17.4)	4 (11.4, 0.3-22.5)	19 (12.8, 7.4-18.3)	- [1]	1.000
**General public**	***n*=311**	***n*=118**	***n*=193**		
Depressive symptoms	141 (45.3, 39.8-50.9)	45 (38.1, 29.2-47.0)	96 (49.7, 42.6-56.9)	3.980	**0.046**
Anxiety symptoms	112 (36.0, 30.6-41.4)	33 (28.0, 19.7-36.2)	79 (40.9, 33.9-47.9)	5.343	**0.021**
Insomnia symptoms	54 (17.4, 13.1-21.6)	19 (16.1, 9.4-22.8)	35 (18.1, 12.7-23.6)	0.211	0.646
PTSS	13 (4.2, 1.9-6.4)	6 (5.1, 1.1-9.1)	7 (3.6, 1.0-6.3)	- [1]	0.568
**All participants**	***n*=494**	***n*=153**	***n*=341**		
Depressive symptoms	236 (47.8, 43.4-52.2)	63 (41.2, 33.3-49.1)	173 (50.7, 45.4-56.1)	3.866	**0.049**
Anxiety symptoms	186 (37.7, 33.4-41.9)	47 (30.7, 23.3-38.1)	139 (40.8, 35.5-46.0)	4.538	**0.033**
Insomnia symptoms	100 (20.2, 16.7-23.8)	32 (20.9, 14.4-27.4)	68 (19.9, 15.7-24.2)	0.062	0.803
PTSS	36 (7.3, 5.0-9.6)	10 (6.5, 2.6-10.5)	26 (7.6, 4.8-10.5)	0.185	0.667

[1] Fisher's Exact Test; Bolded value: <0.05.

Table S2. Prevalence of depressive, anxiety, and insomnia symptoms among participants by severity

Variables	Total *n*=494	Attendant students *n*=183	General public *n*=311
	Number (%)		
Depressive symptoms			
Mild (PHQ-9, 5-9)	185 (37.4)	72 (39.3)	113 (36.3)
Moderate (PHQ-9, 10-14)	34 (6.9)	16 (8.7)	18 (5.8)
Severe (PHQ-9, ≥ 15)	17 (3.4)	7 (3.8)	10 (3.2)
Anxiety symptoms			
Mild (GAD-9, 5-9)	151 (30.6)	54 (29.5)	97 (31.2)
Moderate (GAD-9, 10-14)	23 (4.7)	15 (8.2)	8 (2.6)
Severe (GAD-9, ≥ 15)	12 (2.4)	5 (2.7)	7 (2.3)
Insomnia symptoms			
Mild (ISI, 8-14)	82 (16.6)	36 (19.7)	46 (14.8)
Moderate (ISI, 15-21)	15 (3.0)	7 (3.8)	8 (2.6)
Severe (ISI, ≥ 22)	3 (0.6)	3 (1.6)	0

Figure S1. Scatter plots of the linear correlations between PHQ-9, GAD-7, ISI, fatigue, and PCL-C scores of the general public

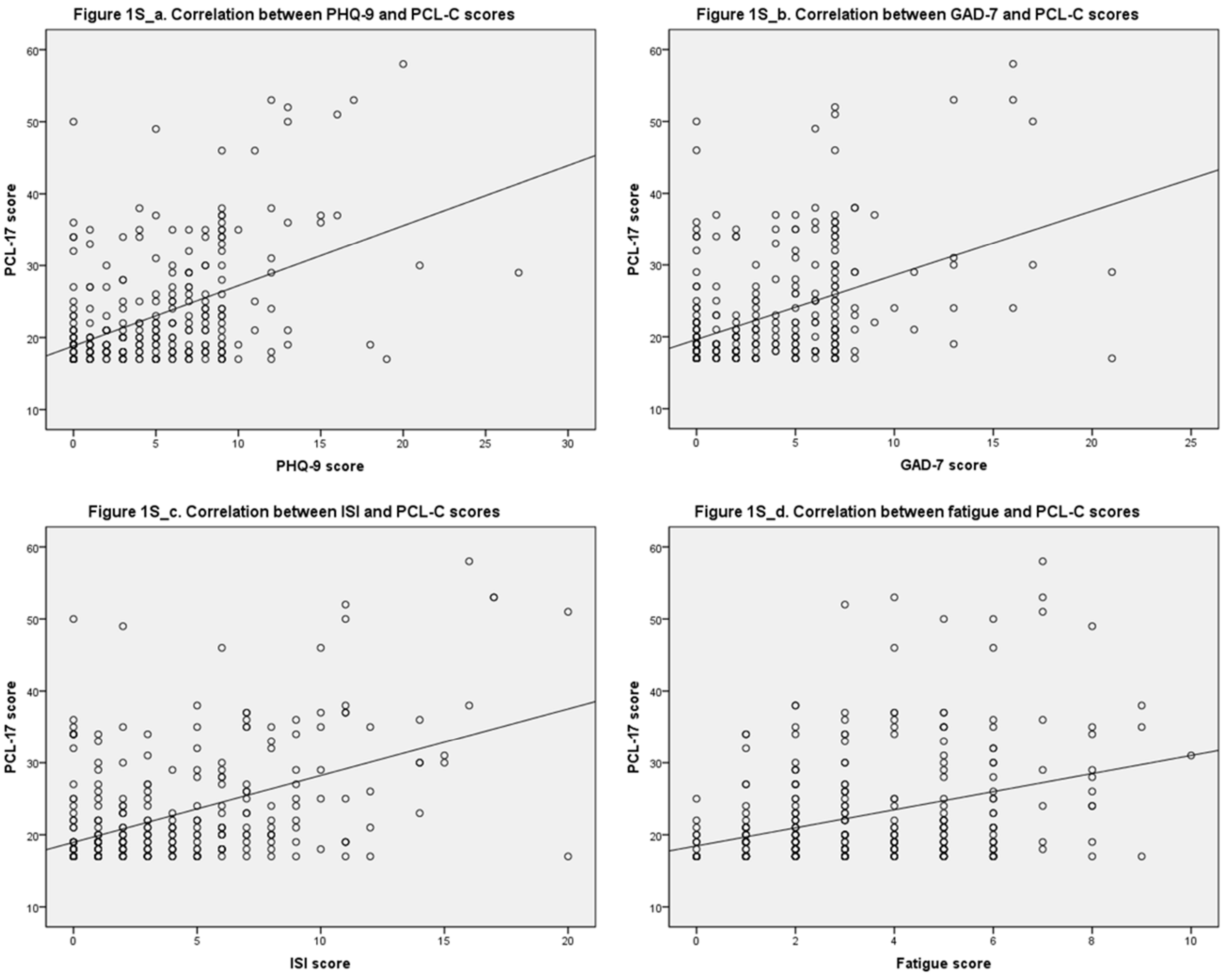

PCL-C score was positively correlated with PHQ-9 score (r = 0.487 for Pearson's correlation; r = 0.470 for Spearman's correlation), GAD-7 score (r = 0.449 for Pearson's correlation; r = 0.469 for Spearman's correlation), ISI score (r = 0.489 for Pearson's correlation; r = 0.434 for Spearman's correlation), and fatigue score (r = 0.351 for Pearson's correlation; r = 0.318 for Spearman's correlation) (all $P < 0.001$).
